# Demineralized Dentin Matrix for Dental and Alveolar Bone Tissues Regeneration: An Innovative Scope Review

**DOI:** 10.1007/s13770-022-00438-4

**Published:** 2022-04-16

**Authors:** Mohammed E. Grawish, Lamyaa M. Grawish, Hala M. Grawish, Mahmoud M. Grawish, Ahmed A. Holiel, Nessma Sultan, Salwa A. El-Negoly

**Affiliations:** 1 Department of Oral Biology, Faculty of Dentistry, Mansoura University, Elgomhouria St., Mansoura, 35516 Egypt; 2 Faculty of Oral and Dental Medicine, Delta University for Science and Technology, Costal International Road in Front of Industrial Area, Mansoura, 11152 Gamasa Egypt; 3 Mansoura Manchester Dental Program, Faculty of Dentistry, Mansoura University, Elgomhouria St., Mansoura, 35516 Egypt; 4 Department of Conservative Dentistry, Faculty of Dentistry, Alexandria University, 22 El-Guish Road, El-Shatby, Alexandria, 21544 Egypt; 5 Department of Dental Biomaterials, Faculty of Dentistry, Mansoura University, Elgomhouria St., Mansoura, 35516 Egypt

**Keywords:** Dentin, Treated dentin matrix, Demineralized dentin matrix, Bone regeneration, Dental tissue regeneration

## Abstract

**Background:**

Dentin is a permeable tubular composite and complex structure, and in weight, it is composed of 20% organic matrix, 10% water, and 70% hydroxyapatite crystalline matrix. Demineralization of dentin with gradient concentrations of ethylene diamine tetraacetic acid, 0.6 N hydrochloric acid, or 2% nitric acid removes a major part of the crystalline apatite and maintains a majority of collagen type I and non-collagenous proteins, which creates an osteoinductive scaffold containing numerous matrix elements and growth factors. Therefore, demineralized dentin should be considered as an excellent naturally-derived bioactive material to enhance dental and alveolar bone tissues regeneration.

**Method:**

The PubMed and Midline databases were searched in October 2021 for the relevant articles on treated dentin matrix (TDM)/demineralized dentin matrix (DDM) and their potential roles in tissue regeneration.

**Results:**

Several studies with different study designs evaluating the effect of TDM/DDM on dental and bone tissues regeneration were found. TDM/DDM was obtained from human or animal sources and processed in different forms (particles, liquid extract, hydrogel, and paste) and different shapes (sheets, slices, disc-shaped, root-shaped, and barrier membranes), with variable sizes measured in micrometers or millimeters, demineralized with different protocols regarding the concentration of demineralizing agents and exposure time, and then sterilized and preserved with different techniques. In the act of biomimetic acellular material, TDM/DDM was used for the regeneration of the dentin-pulp complex through direct pulp capping technique, and it was found to possess the ability to activate the odontogenic differentiation of stem cells resident in the pulp tissues and induce reparative dentin formation. TDM/DDM was also considered for alveolar ridge and maxillary sinus floor augmentations, socket preservation, furcation perforation repair, guided bone, and bioroot regenerations as well as bone and cartilage healing.

**Conclusion:**

To our knowledge, there are no standard procedures to adopt a specific form for a specific purpose; therefore, future studies are required to come up with a well-characterized TDM/DDM for each specific application. Likely as decellularized dermal matrix and prospectively, if the TDM/DDM is supplied in proper consistency, forms, and in different sizes with good biological properties, it can be used efficiently instead of some widely-used regenerative biomaterials.

**Supplementary Information:**

The online version contains supplementary material available at 10.1007/s13770-022-00438-4.

## Introduction

**Fig. 1:**
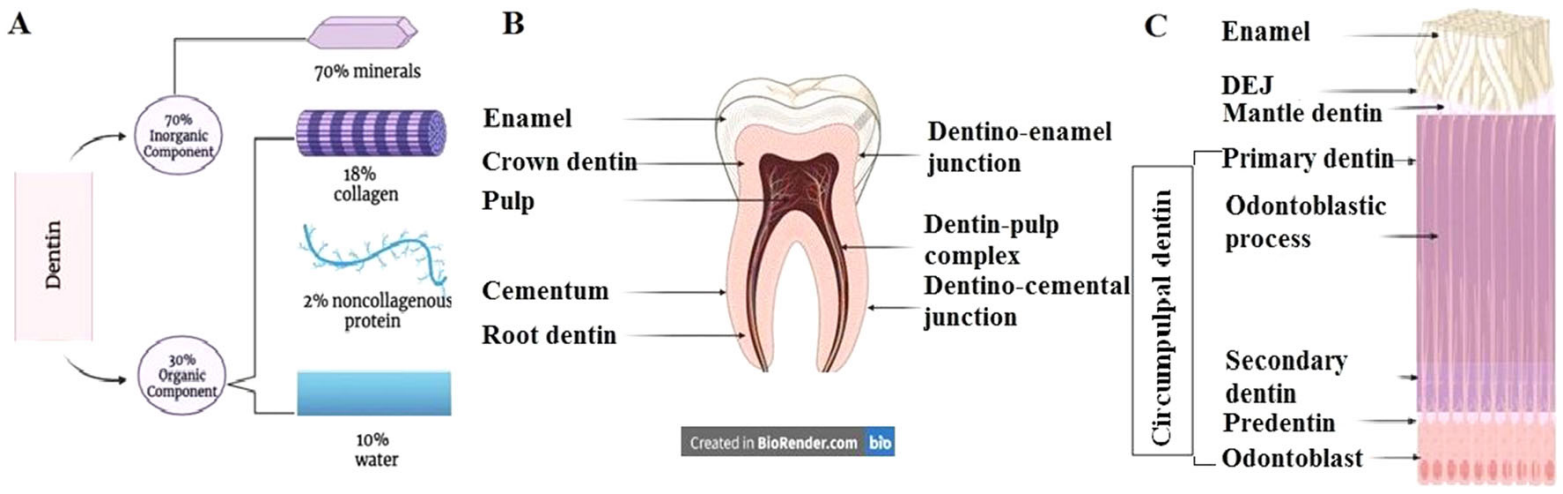
Representative diagrams created with BioRender.com for the chemical composition of **A** dentin, **B** component parts of the tooth and **C** histological structure of dentin

Dentin is chemically composed of approximately 70% mineral phase (40%–45% in vol), 20% organic matrix (30% in vol), and 10% water (20–25% in vol). Additionally, the organic component of dentin consists of 18% collagen and 2% noncollagenous proteins (NCP), proteoglycans, growth factors, phospholipids, and enzymes (Fig. 1A). The matrix is a repository for growth factors, such as basic fibroblast growth factor, insulin-like growth factor, transforming growth factor-β, and bone morphogenetic proteins (BMP). Several NCPs, such as osteopontin and osteocalcin, are common in dentin and bone; however, dentin phosphoprotein is an NCP found specifically in dentin [1]. The inorganic components of dentin are calcium and phosphate ions that form hydroxyapatite crystals that are larger compared with those found in bone and much smaller than those in enamel [2].

Regarding the histological structure of dentin, it is considered as a specialized mineralized avascular connective tissue that forms the main bulk of the tooth. It is covered by enamel on the crown and cementum on the root and surrounds the entire pulp tissue (Fig. 1B). Beneath the enamel, dentin has an outer mantle layer of 15–30 µm thickness whereas underneath the cementum, Tomes granular and/or the hyaline Hopewell-Smith layers are identified, and each of them represents approximately 15–30 µm thickness. The circumpulpal dentin forms the main bulk of the dentin, and its thickness continuously increases by about 4 µm/day at the expense of dental pulp space. Circumpulpal dentin includes the intertubular dentin and peritubular (intratubular) one. Compared with intertubular dentin, peritubular dentin has a relatively higher proportion of sulfated proteoglycans and minerals with lesser collagen fibrils; therefore, it is considered harder than intertubular dentin. Intertubular dentin results from the transformation of predentin into dentin and it is a composite consisting of collagen fibrils discontinuously reinforced with nanoplates of carbonated hydroxyapatite [3].

Dentin is highly permeable as it contains numerous dentinal tubules running from the pulp tissue to the dentino-enamel junction (DEJ) in the crown and till the dentino-cemental junction (DCJ) in the root. Dentin exhibits regional differences in tubule density and diameter wherein tubule diameter can vary from 0.9 µm peripherally to 2.5 µm at the pulp side. In the meantime, density is approximately 59,000–76,000 tubules/mm^2^ at the pulp side whereas the number of these tubules decreases to half of such quantity at the area close to the DEJ. Dentinal tubules have collateral branches measuring 1 μm in diameter that form a three-dimensional network as they extended at specific angles crisscrossing intertubular dentin [4].

The dentinal tubule contains an odontoblast cell process, which is an extension of an odontoblast cell, and serum-like fluid which contains a mixture of proteoglycans, tenascin, transferrin, and albumin. Interestingly, odontoblast processes were seen only in the tubules near the pulp. Further, odontoblasts differentiate from ectomesenchymal cells of the dental papilla and are organized at the periphery of the pulp as a cellular palisade. They form the dentin matrix (predentin) by synthesizing collagen types I, III, and V; noncollagenous proteins as integrin-binding sialoprotein, matrix extracellular phosphoglycoprotein, osteopontin, dentin matrix protein 1, and sialophosphoprotein; glycoproteins as dermatan sulfate, keratan sulfate, heparan sulfate, and chondroitin sulfate [5]. They are also responsible for the deposition of minerals in the dentin matrix, which is not simply restricted in the mineralization front at the edge of predentin and dentin but occurs along the whole length of the odontoblast process [6].

During tooth development and organogenesis, odontoblasts form the primary dentin which comprises the main bulk of the circumpulpal dentin. After root completion and throughout the life of the tooth, odontoblasts form secondary dentin bordering the pulp at a slow rate (Fig. 1C). This type of dentin contains fewer dentinal tubules than primary dentin and there is usually a bend in the tubules at the interface between primary and secondary dentin. In response to environmental conditions and according to the severity of the stimuli, tertiary dentin (reactionary/reparative) is formed as odontoblasts forming the reactionary dentin while the dental pulp stem cells form the reparative one. Sometimes, the word synonyms of tertiary, reactionary, and reparative terms are used interchangeably [7].

At the micron length scale, crown dentin is similar to root dentin; however, unlike root dentin, the proportion of the tubular area is higher and tubules follow a gentle S-shaped curve in the crown part while they are straight in the root area. The dentinal tubules are surrounded by a 2–6 µm dense cuff of peritubular dentin, which suggests that the dentin mineral density is higher in the crown than in the root and the predentin is significantly wider in the crown than in the root [8].

Considering the physical and biological properties of dentin, the thickness is approximately 3–10 mm or even more, and this thickness differs in the regional parts of the same tooth, among different teeth and as a result of aging. The color of dentin greatly affects the color of the tooth due to the translucency of enamel as dentin is a yellowish-hued material that becomes darker with age. In radiographic images, dentin is more radiolucent than enamel due to its lower mineral content whereas it is more radiopaque than cementum and bone. Dentin has no replacement mechanism for biologic turnover but it can be remodeled to a certain degree as odontoblasts and pulpal resident stem cells can produce secondary and tertiary dentin when it is damaged by excessive tooth wear, carious lesion, trauma, or through iatrogenic insult, such as accidental exposure. Dentin is a bone-like matrix that is a vital, sensitive and porous tissue, capable of responding to mechanical, thermal, chemical, evaporative, and osmotic environmental stimuli. Dentin can be regarded as both a barrier and permeable structure, depending upon its thickness, age, and other variables. The permeability to fluid flow through the dentinal tubules, as well as a directional design of these tubules, suggests that dentin has a sensory hydrodynamic function. Micropore-sized dentinal tubules that measure 0.9–2.5 µm diameter provide micropore spaces of 3.70%–5.88% porosity that increases the surface contact area of dentin [3].

Regarding the mechanical properties of dentin, the elastic modulus and hardness gradually increase from the pulp side toward DEJ and DCJ. The poorly mineralized intertubular dentin has a lower Young's modulus than the highly mineralized peritubular dentin. A hydrated environment affects the mechanical behavior of dentin, as the elastic modulus decreases by 35% and hardness decreases by 30% [9]. Young dentin has higher initial toughness and stable toughness values than aged dentin. The fatigue crack growth exponent is associated with the direction of the dentinal tubules [10]. Unlike enamel, dentin is less brittle and somewhat has viscoelastic properties. This elasticity is important to provide the flexibility that is required to support the overlying enamel and prevent its fracture. The tensile strength of dentin is attributed to the fibrous arrays of collagen type I, while high compressive strength and rigidity are provided by the crystals of the mineralized phase deposited within the collagen fibers [7].

To obtain tooth-derived substances, demineralization is required before clinical adaptation to open the dentinal tubules and release BMPs. Demineralization is the process of removing some of the highly crystalline inorganic substances from the dental hard tissues, which results in the loss of its structural integrity with the collapse and degradation of the supporting collagen matrix. Enamel and dentin contain calcium-deficient carbonate-rich hydroxyapatite crystallites with enamel having much larger crystals compared with dentin. The crystal sizes comprise 85 vol% of enamel structure compared to about 50 vol% in dentin. The larger surface area of the dentin apatite increases its dissolution susceptibility when exposed to acids and this susceptibility is further increased because of the higher carbonate content of the dentin mineral. Thus, the dentin minerals are dissolved more rapidly than the enamel ones, and because there is less total mineral in dentin than in enamel, the acid attack proceeds more quickly in dentin [11].

By preferentially removing peritubular dentin, acid-etching agents used during dental restorative procedures and ethylenediamine tetraacetic acid (EDTA) used in endodontic treatments enlarge the openings of the dentinal tubules, making the dentin more permeable. Specific acids and chelating agents as 17% EDTA, sodium hypochlorite, and citric acid were used during root canal instrumentation to remove the smear layer and clean dentinal walls [11]. Moreover, bacterial acids (lactic acid), dietary acids (acetic acid, phosphoric acid, and citric acid), and gastric acid {hydrochloric acid (HCl)}, all demineralized the dentin through the processes of caries and acid-erosion. It has become standard to use laboratory acids, such as formic acid [12, acetic and lactic acids [13], or chelating agents such as EDTA [14], to produce demineralized dentin models for use in *in vitro* remineralization studies. It is well known that each demineralizing agent has a unique effect as chelating agents, strong acids, and weak acids affecting both mineral and organic phases of dentin in significantly different ways. For example, the demineralizing agents caused some degree of collagen denaturation, citric acid caused the most damage and varying the concentrations of EDTA and citric acid affected collagen in a dissimilar manner [15].

In the past decade, teeth as graft material have been proposed with fascinating outcomes. Therefore, this study aimed to review evidence on treated dentin matrix (TDM)/demineralized dentin matrix (DDM) for dental and bone tissues regeneration and summarize the *in vitro* and *in vivo* animal and human studies using TDM/DDM as an osteoinductive material for clinical applications.

## Materials and methods

### Study design

We conducted a scoping review and searching for articles on TDM/DDM for tissue regeneration. For our scoping review, a five-stage framework was adopted following Arskey and O'Malley's design [16]. The five stages were: specifying research questions; identifying relevant studies; studies selection; extraction, mapping and charting the data; collating, summarizing, synthesizing and reporting results.

#### Stage I: identification of research questions

We aimed to answer the following questions; (1) is there is a difference between TDM and DDM, (2) with respect to species and tooth parts, what are the different sources of obtaining TDM/DDM, (3) what is the conventional forms and shapes of TDM/DDM and their sizes, (4) what is the most widely-used demineralizing agents and what is the optimal exposure time, and (5) what are the most common preclinical and clinical applications of TDM/DDM in tissue regeneration.

#### Stage II: identification of relevant studies

**Fig. 2:**
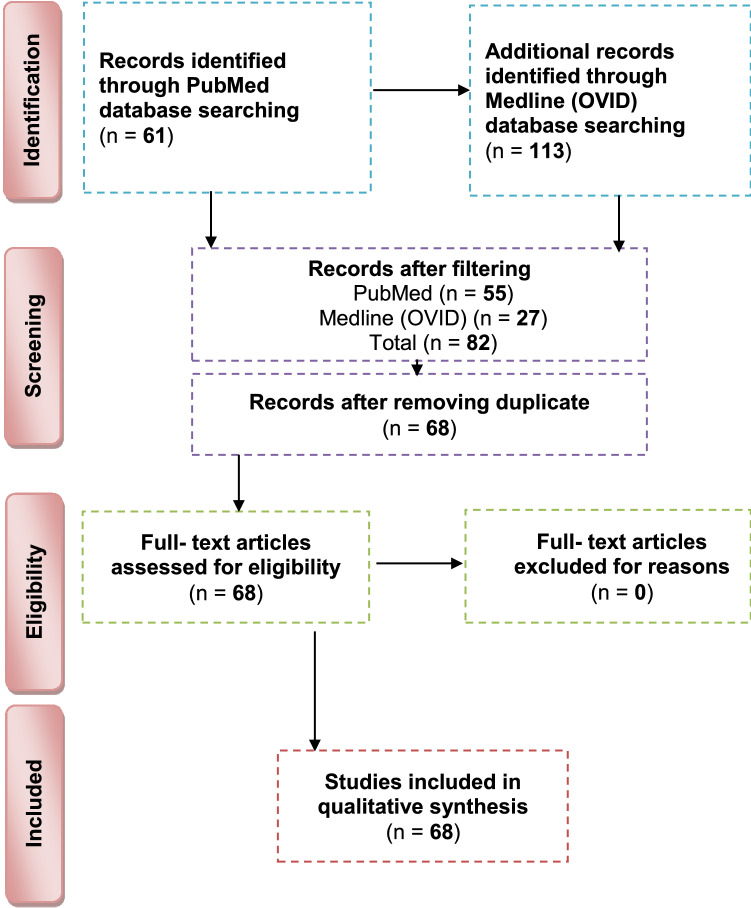
Flowchart for article selection according to preferred reporting items for systematic reviews and meta-analyses guidelines

PubMed and Medline (OVID) databases were searched in October 2021 for the relevant articles performed on TDM/DDM using the following strategy for PubMed search {(treated dentin matrix [Title/Abstract]) AND (regeneration [Title/Abstract])/(demineralized dentin matrix [Title/Abstract]) AND (regeneration [Title/Abstract])} and the following one for Medline (OVID) databases)treated dentin matrix and regeneration).af (all fields) and (demineralized dentin matrix and regeneration).af. Figure 2 demonstrates the studies’ search procedure using the PRISMA flowchart.

#### Stage III: selection of studies

The relevant studies were selected according to the inclusion and exclusion criteria. The searches were not restricted by language type, however, were limited to original researches including *in vitro* and *in vivo* animal and human studies and excluding narrative reviews, systematic reviews, and meta-analyses.

#### Stage IV: extraction, mapping, and charting the data

A template was established and reviewed by each author. The authors were calibrated to extract the following data; authors, publication year, country of origin, study design, source of TDM/DDM (human or animal), tooth part (crown or root), matrix form, matrix size, demineralization protocol, ways of sterilization, and preservation and the outcome.

#### Stage V: collating, summarizing, synthesizing, and reporting results

Meta-synthesis and integration of findings from qualitative studies were performed to provide a new and more comprehensive interpretation of the findings.

### Statistical analysis

Degree of chance–adjusted agreement (kappa coefficient value) was used to determine the inter-reviewer reliability.

## Results

### Studies' selection and distribution of relevant articles according to date of publication, country origin and study designs

The initial search identified 113 unique references. No additional studies were recognized through hand searching. After filtering, 82 references were recorded and screened. After the eligibility criteria were applied and duplicates were removed, 68 *in vitro*, *in vivo* animal and human studies were obtained and were included in the present review (Fig. 2). The kappa value for inter-reviewer agreement was 0.85. The distribution of relevant articles according to the date of publication, distribution of relevant articles according to country of origin, study designs of relevant articles, teeth involved in study designs, tooth part involved in study designs, forms and sizes, demineralization protocol, methods of sterilization, and preservation and outcome are presented in Supplementary Tables 1, 2, 3, and 4.

Considering publication year, the highest number of articles was published in 2021 (19.11%) whereas the lowest ones were published in 2014 (1.47%), 2008 (1.47%), 2005 (1.47%), 2002 (1.47%), and 2001 (1.47%). The percentage of articles published in 2020, 2019, 2018, 2017, 2016, 2015, 2013, 2012, 2011, and 2009 were 8.82%, 5.88%, 8.82%, 10.29%, 10.29%, 13.23%, 5.88%, 4.41%, 2.94%, and 2.49%, respectively. No articles were published in 2010, 2007, 2006, 2004, and 2003. Regarding the country of origin, most of the studies were performed in China (50%), Republic of Korea (16.17%), Japan (10.29%), Brazil (7.35%), Iran (4.41%), Egypt (2.94%), Sweden (2.49%), Taiwan (1.47%), UK (1.47%), USA (1.47%), and Thailand (1.47%). The study designs were *in vitro*, *in vivo* animal studies, case reports, *in vivo* human studies, randomized controlled clinical trials, and split-mouth randomized controlled clinical trials.

### Answering research questions

**Fig. 3:**
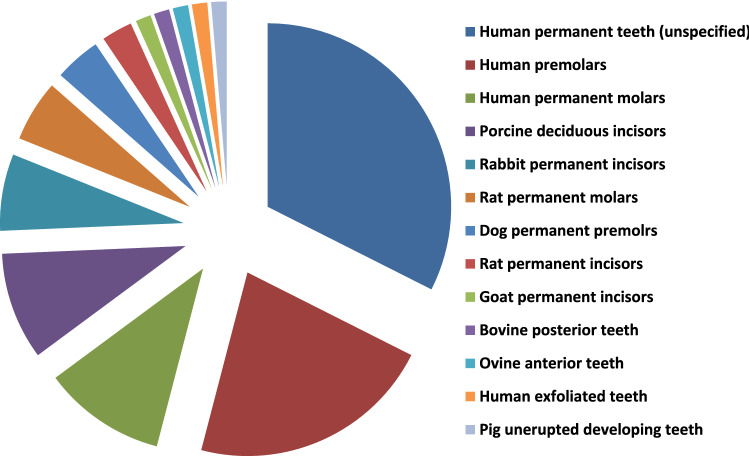
Exploded pie chart showing analytical data of the frequencies regarding source of teeth selected in study designs from the relevant articles

**Fig. 4:**
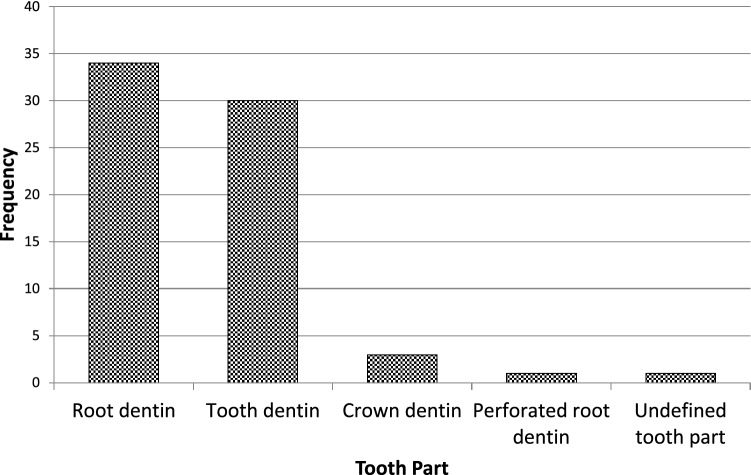
Bar chart showing analytical data of the frequencies regarding tooth part selected in study designs from the relevant articles

**Fig. 5:**
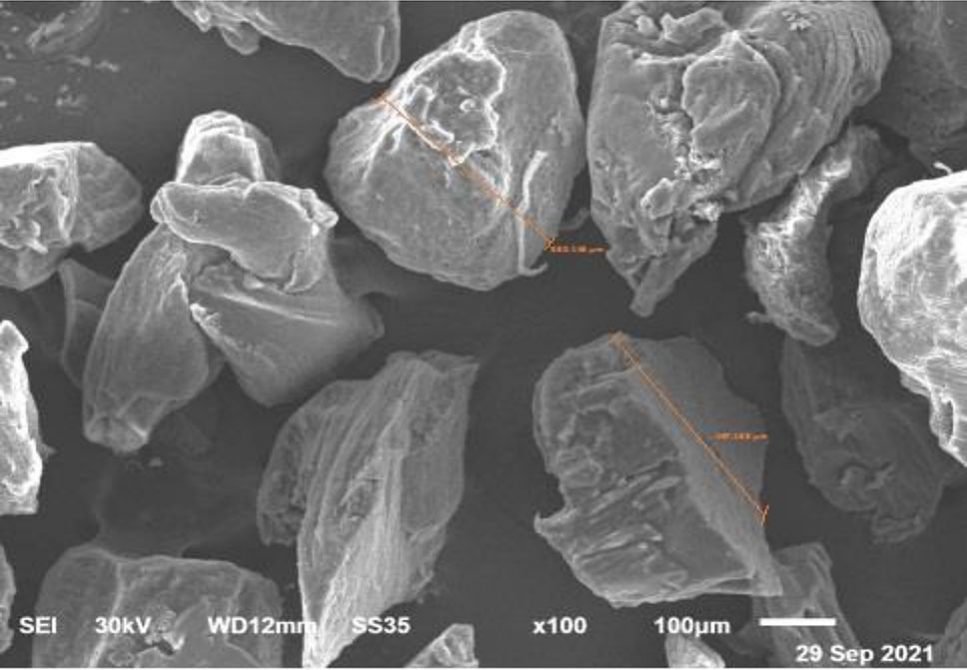
SEM images showing DDM particle size ranging from 350-500lm. Courtesy provided by the staff members of Oral Biology, Faculty of Dentistry, Mansoura University, Mansoura, Egypt

**Fig. 6:**
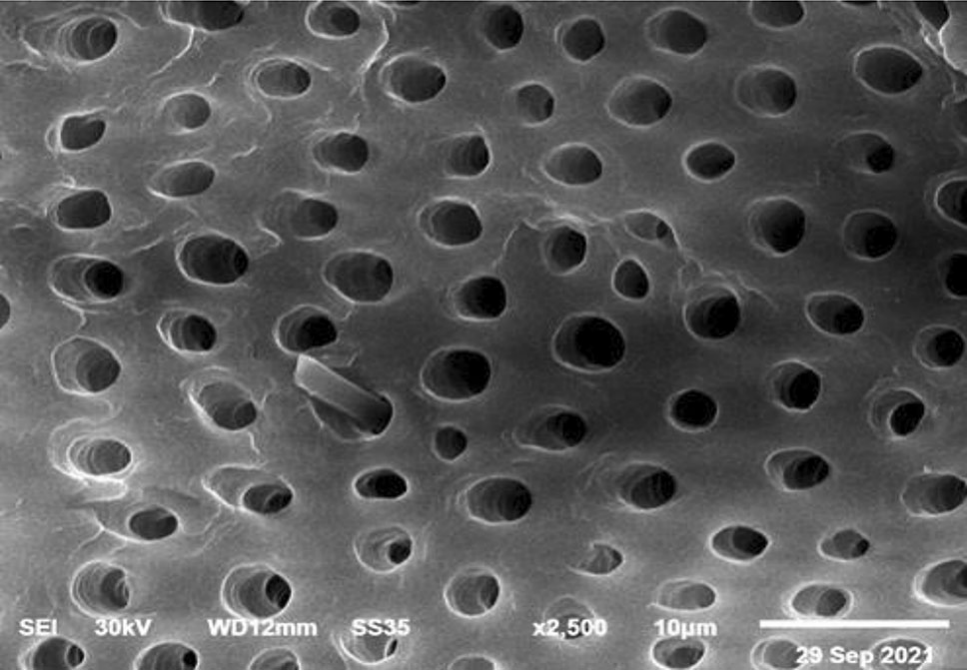
SEM images showing the basic dentin micro-texture after demineralization. Structurally, dentinal tubules are enlarged. Courtesy provided by the staff members of Oral Biology, Faculty of Dentistry, Mansoura University, Mansoura, Egypt.

**Fig. 7:**
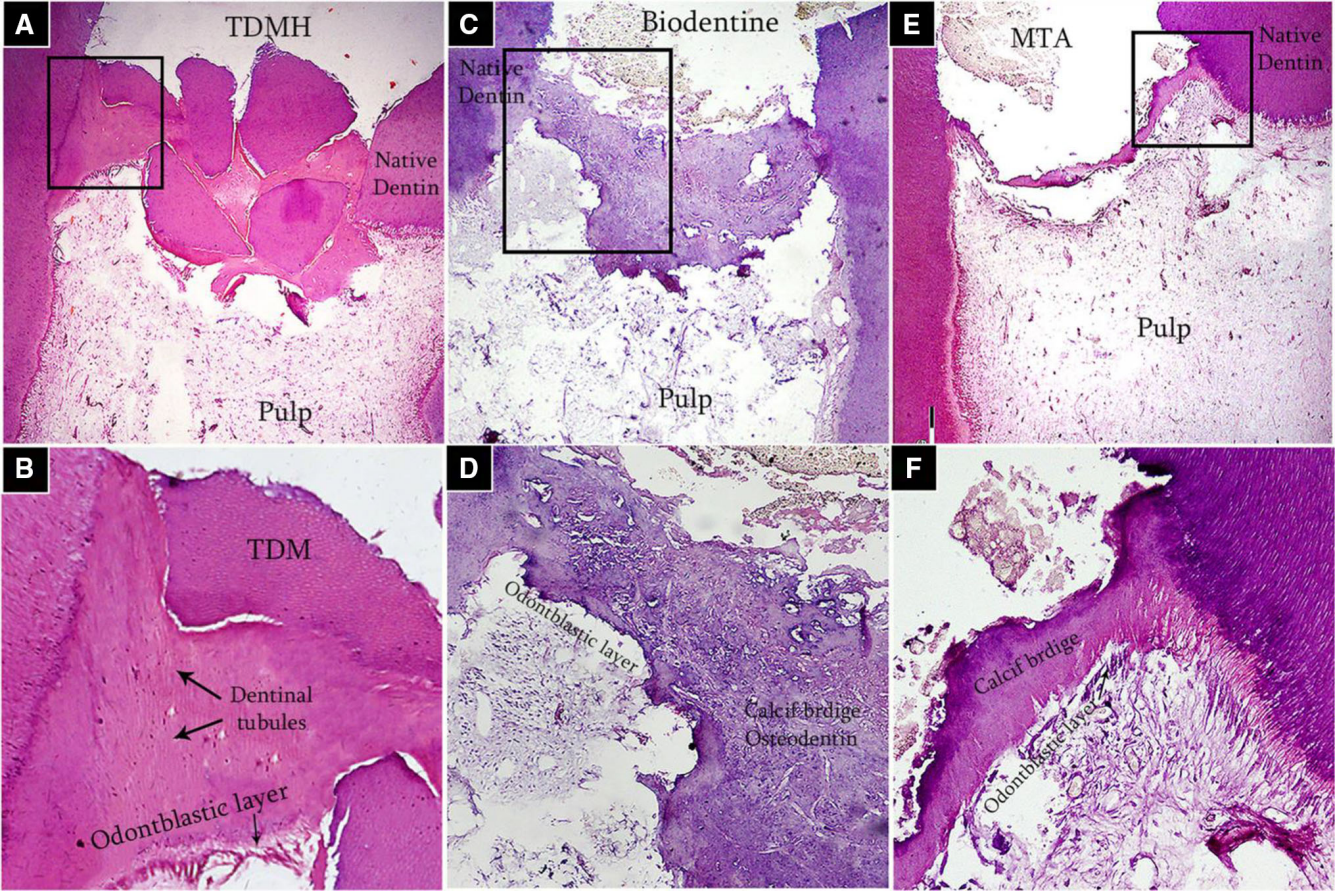
Decalcified sections of human pulp capped with **A**, **B** DDM hydrogel, **C**, **D** Biodentine, and **E**, **F** MTA after 2-mon examination period showing complete dentin bridge formation and absence of inflammatory pulp response. a, b, c × 40 and a1, b1, c1 are higher magnification of boxed areas × 200. Courtesy provided by the staff members of Conservative Dentistry, Faculty of Dentistry, Alexandria University, Alexandria, Egypt

**Fig. 8:**
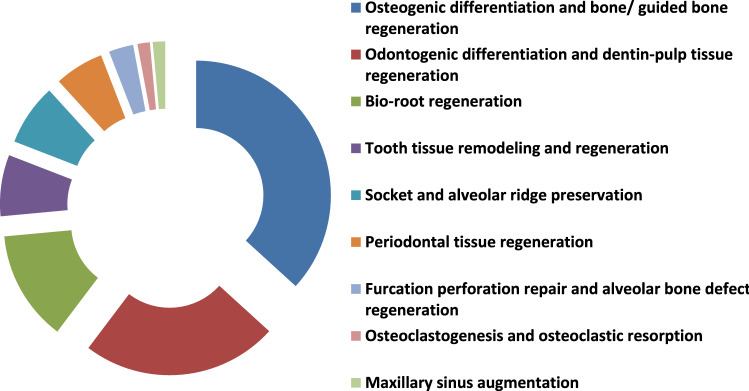
Exploded doughnut chart showing outcomes from the relevant articles

1. **Is there a difference between TDM and DDM?**
  The search strategy of PubMed and Medline (OVID) databases using the appropriate search terms yielded 55 articles for PubMed database and 13 articles for Midline (OVID). Among them, there were 32 articles for TDM and 36 articles for DDM. Both terms were used interchangeably despite that the matrices were fabricated with the same protocols using gradient concentrations of EDTA, 0.6 N-HCl or 2% HNO_3_. In our opinion, it is devisable to use the term DDM for the dentin matrices prepared for regenerative purposes that obtained from different sources, fabricated in different forms and shapes, and demineralized with different protocols as the term TDD could mean physical treatment using laser therapy or mechanical one by means of abrasives.
2. **Teeth and tooth parts involved in study designs**
  The majority of the published studies relied on harvesting vital and nonvital human permanent teeth. The human teeth were maxillary and mandibular premolars extracted for orthodontic reasons, nonfunctional third molars requiring removal for clinical reasons and exfoliated deciduous teeth. In addition, teeth obtained from other animal species were involved in a few study designs such as porcine deciduous incisors, rabbit permanent incisors, rat permanent molars, dog permanent premolars and rat permanent incisors, that were frequently selected whereas goat incisors, bovine posterior teeth, ovine lower anterior teeth and pig unerupted developing teeth were selected to less comparatively (Fig. 3).
  Primarily and with the use of proper dental instruments, enamel, cementum, and periodontal tissues of the extracted teeth were completely removed from the outer tooth surface, and pulp tissue with predentin was completely removed from the internal one. Root dentin is the tooth part that is commonly used whereas crown dentin was used to a lesser degree (Fig. 4).
3. **Forms and sizes**
  The TDM/DDM were produced in different forms likely as particles, liquid extract, hydrogel, and paste, and in different shapes such as sheets, slices, chips, blocks, discs, root-shaped, barrier membranes, and press-fitted according to the defect shape.
  Dentin grinder and ball mill machines were used for preparing particulate dentin [17, 19, 22, 25, 26, 45, 49–52, 55, 56–60, 62, 65, 68, 73, 74, 76, 77, 80]. The diameter of the particle size was measured in either µm or mm. The minimum diameter was less than 40 µm [74] whereas the maximum was 2 mm [45, 58, 59]. In certain instances, the particles were atelopeptidized [25], decorated with carboxymethyl chitosan [49], loaded with recombinant human bone morphogenic protein-2 [52], encapsulated into liposomes [54], or used as particle-based bio-ink [47] (Fig. 5). An extract [19, 22, 54, 63, 74] was obtained through soaking particles of 1 g in 5 ml saline [19], adding particles of 20 g to 100 ml DMEM/F12 and filtered using a 0.22 μm filter [22], reconstituting particles of 0.5 g to 1 mg/ml tris-buffered saline and sterile filtered through a 0.22-µm filter [54], or by adding granules of 10 mg to 1 ml α-MEM and then the extract was diluted to 1 mg/ml, or used as 10 mg/ml [63]. Hydrogel [21] was prepared by dispersing particles measuring 350–500 µm in 0.125 g sodium alginate solution in a 1:1 ratio and then dripped into 5% (w/v) sterile CaCl_2_ solution. The paste form [29] was prepared by mixing a powder of DDM with particle size less than 76 µm to aqueous extract of DDM with a volume ratio of 1:1.
  Sheets [19, 23, 42, 44, 78] were sized in 2 × 6 × 6 mm matrices [23, 42, 44] or fabricated in a porous shape of 100 µm thickness [78], and in certain instances, the sheets were autoclaved [23]. Slices [61, 69–71, 82] were 8 µm thickness [61, 69–71], chips were 1 mm thickness [75], and blocks [79, 81, 83] were 5 mm diameter and 2 mm thickness [79] or 5 mm diameter and 2 mm height [81] or 2–3 mm thickness and 4 mm diameter [83] whereas discs were 5 mm thickness [24]. The sizes of the root-shaped matrix [18, 20, 27, 28, 30, 32, 34–38, 41, 43, 48] for human premolars were 10 mm in length and 1.0 mm in thickness [18, 32, 41] or 10 mm in length and 3-5 in mm diameter [38]. Regarding molars, the average length and width were 13.08 and 8.41 mm, respectively, and then perforated with uniformly distributed thirty pores measuring 1 mm in diameter [48]. Root-shaped prepared from porcine deciduous incisors [18, 28, 30, 34] were also prepared with the following sets of dimensions: 10 mm length and 1.0 mm thickness [18], 10 mm length and 3–5 mm diameter [28, 30], 2 mm length and 1 mm diameter [28] or in 9.4 mm length with 4.9 mm upper diameter and 3.4 mm bottom diameter [34], and in addition, tubes of 8 mm length were prepared from rat incisors [84]. Barrier membrane size was 300–800 µm thick slice with 0.2–0.3 mm diameter holes [46] or it was produced in the form of semi-rigid cubic shape with 2 mm × 2 mm × 8 mm [64]. Finally, the DDM was also manufactured to be press-fitted to the premolar furcation defect with a 2-mm diameter scaffold [31].
4. **Methods of demineralization, sterilization and preservation**
  To our knowledge, no standard protocol for demineralization was established for the different forms and shapes of dentin matrices used for tissue regeneration. Therefore, the protocol requires optimization of the concentration and pH of the demineralizing agent, and the exposure time needs to be properly adjusted for each form and shape. Before demineralization, it is necessary to prepare dentin matrix to remove the debris resulting from mechanical instrumentation. Accordingly, the dentin matrices were soaked in deionized water for 5–6 h and a cleaning cycle of 5–20 min was performed every hour using an ultrasonic cleaner [34, 40, 43]. There are several demineralizing protocols tested with different study designs. Excluding the size parameter of the dentin matrix, the particles obtained from human permanent teeth were demineralized with consecutive gradient concentrations of 17%, 10%, and 5% EDTA [17, 26, 49] with different time frames. Correspondingly they were 10 min, 5 min, and 10 min [17], 10 min, 10 min, and 10 min [26], and 5 min, 5 min, and 10 min [49]. In addition, 0.6 N HCl [45, 50, 52, 57, 65, 68, 73, 77, 80] was used for demineralization and the exposure time was considered once and varied between 15 min [77], 30 min [52, 73] and 12 h [50]. The particles obtained from human exfoliated deciduous teeth were demineralized once with 0.6 N HCl and exposure time was varied between 10 min, 15 min, 20 min, 25 min, 30 min, 60 min, and 90 min [80]. Moreover, 2% HNO_3_ [51**, **53**, **55**, **62] was also used for demineralization with two period's time frame, 10 min [55] or 20 min [53]. Similarly, particles obtained from bovine posterior teeth were demineralized using gradient concentrations of 17% EDTA for 1, 7 and 13 days [25] each. Alternatively, particles obtained from rabbit mandibular incisors [65] and ovine lower anterior teeth [68] were demineralized with 0.6 N HCl once and the exposure time was 7 days for the whole tooth before pulverization to small particles whereas particles obtained from rat incisors were demineralized using 17% EDTA for 10 min [67].
  Human-derived particles used for preparing liquid extracts were demineralized with 17%, 10%, and 5% EDTA with two different time frames as 5 min, 5 min, 10 min [22], 30 min, 30 min, 30 min [74] and in addition, porcine-derived particles used for the same purpose were exposed for 30 min each [74]. Hydrogel was prepared by incorporating human-derived particles demineralized with 17%, 10%, and 5% EDTA for 10 min, 10 min and 5 min [21], respectively, in sodium alginate solution. Moreover, human and porcine-derived particles used to prepare paste form were demineralized with 17%, 10%, and 5% EDTA for 10 min, 10 min, and 5 min [29], respectively.
  Human-derived sheet scaffolds were demineralized with 17% and 5% with two different time frames as 5 min, 5 min [23] and 4 min, 2 min [42, 44] or alternatively demineralized with 0.6 N HCl for 2 weeks [78] while human-derived slices were demineralized with 10% EDTA for approximately 3 months [82]. The human-derived dentin chips were demineralized with 17%, 10%, and 5% for 5 min, 5 min, and 10 min [75], respectively. Also, human-derived dentin blocks were demineralized with 24% EDTA for 12 h [79] and blocks derived from unerupted developing pigs' teeth were demineralized with 24% EDTA for 2 min, 6 min, and 12 min [83], respectively. The human-derived disc-shaped scaffolds were demineralized with 10% EDTA for 3 days, and then successively soaked in 17%, 10%, and 5% for 20 min, 20 min, and 20 min [24], respectively. Demineralization of the root-shaped matrix obtained from human sources was performed with 17%, 10%, and 5% EDTA for 5 min, 5 min, and 10 min [18, 32, 35, 38, 41, 43] respectively, or for 12 min, 12 min, 20 min [27], for 30 min, 30 min, 30 min, respectively, or demineralized with 0.34 N HNO_3_ for 30 min [48] whereas those obtained from porcine deciduous incisors were demineralized with 17%, 10%, and 5% EDTA for 20 min, 18 min, and 15 min [18, 28, 30] or for 20 min, 20 min, 10 min [34], or for 10 min, 10 min, 5 min [36], respectively, and those obtained from dogs were demineralized with 17%, 10%, and 5% for 12 min, 12 min, and 20 min [20], or for 8 min, 8 min, 12 min [37], respectively. Dentin tubes obtained from rat incisors were demineralized with 0.6 N HCl for 3 h [84]. Human-derived barrier membranes were demineralized with 0.6 N HCl [46]. Scaffolds prepared to be press-fitted into dogs' furcation perforation were demineralized with 17%, 10%, 5% for 5 min, 5 min, 10 min [31], respectively. Figure 6 presents DDM with opened dentinal tubules after demineralization.
  Sterilization was performed by maintaining the matrices in sterile phosphate-buffered saline (PBS) with 100 units/ml of penicillin and 100 μg/ml of streptomycin at 37 °C, for 72 h and then washed in sterilized deionized water for 5 min [17, 21, 22, 24, 28, 30–36, 38–44], socking into penicillin and streptomycin [72] only, preservation in 5% peracetic acid and 75% ethanol for 10 min [53, 80, 81], immersion in 5 ml alcohol/2 ml of gentamicin [61, 69–71], rinsing with sterile saline for 10 min [75], washing in sterilized deionized water for 10 min [82], rinsing twice in 0.1 M Tris-HCl (pH 7.4) for 10 min [55, 62] and using gradient ethanol concentrations [47]. Other methods of sterilization were gamma irradiation processing using Cobalt 60 radiation with a dose of 5 kGy [66], ethylene oxide gas sterilization at low temperatures [19, 45, 49, 52, 57, 58, 60, 67, 68, 76], steam sterilization at 121°C and a pressure of 1 bar for 15 min [23], lyophilization and freeze-drying [46], or mixing was accomplished in a sterile container [65].
  The matrices were preserved in α-MEM media containing 50 units/ml of penicillin and 50 μg/ml of streptomycin or in 0.1 x PBS and kept in refrigerator at 4 °C [18, 20, 24, 27, 28, 30–32, 34, 36, 39–42, 44, 47] cryopreserved at -196°C [38], −80 °C [26], −18 °C [83], and -20°C [25] or stored at room temperature [45, 52, 65]. Other methods of preservation included syringes for hydrogel [21], package for barrier membranes [46] and particles [58–60], or freshly prepared during operation [53, 55, 63]. In addition, slices were stored at 2°C until implantation [61, 69–71].
5. **Outcome**
  The TDM/DDM were considered for osteogenic differentiation [39, 49, 50] and bone/guided bone regeneration [45, 46, 49, 51, 53, 55–62, 66, 69–71, 80–82, 84], odontogenic differentiation [17, 40], and dentin-pulp tissue regeneration [25, 38] (Fig. 7), bio-root regeneration [18, 24, 26, 27, 34], tooth tissue remodeling and regeneration [30], socket preservation and alveolar ridge augmentation [52, 55, 73, 76, 82], periodontal tissue regeneration [32, 64, 78], furcation perforation repair [31] and alveolar bone defect regeneration [57], osteoclastogenesis and osteoclastic resorption [28], and maxillary sinus augmentation [55, 77] (Fig. 8).
  Regarding dentin-pulp complex regeneration, Holiel et al. [21] evaluated clinically the regenerative potential of DDM hydrogel as a direct pulp capping material in comparison with Biodentine and mineral trioxide aggregate (MTA). The study was performed on thirty intact fully erupted premolars scheduled to be extracted for orthodontic reasons and they found that hydrogel could achieve dentin regeneration and conserve pulp vitality and might serve as a reasonable natural substitute for Biodentine and MTA in restoring *in vivo* dentin defects. In addition, Mehrvarzfar et al. [75] compared in a clinical trial of thrity-three intact third molars of eleven healthy volunteers the pulpal responses to MTA and combination therapy of MTA and DDM as a pulp-dressing agent s for partial pulpotomy. They found that the dentin bridge was significantly thicker in MTA/DDM group than MTA group alone.
  Considering alveolar bone regeneration, Um et al. [52] evaluated in a case series study the efficacy of DDM loaded with recombinant human bone morphogenetic protein-2 (rhBMP-2) on ten experimental sites for socket preservation. They suggested that DDM may be a potential carrier for rhBMP-2 and it may be conceivable to reduce the concentration of rhBMP-2 to 0.2 mg/ml. In addition, Li et al. [53] conducted a clinical prospective study on forty patients and concluded that autogenous granules of DDM prepared at the chair side after extractions could act as an outstanding readily available alternative to bone graft material in guided bone regeneration, even for implantation of severe periodontitis cases. Moreover, Minamizato et al. [55] evaluated in a pilot study of sixteen patients underwent dental implant placement the clinical application of autogenous partially DDM prepared immediately after extraction for alveolar bone regeneration in implant dentistry and they considered partially DDM as an efficient, safe, and reasonable bone substitute. Furthermore, Pang et al. [57] conducted a prospective randomized clinical trial of a total 33thrity-three graft sites in twenty-four patients and they suggested that autogenous DDM is a viable option for alveolar bone augmentation following dental extraction, in comparison with anorganic bovine bone. Likewise, Elfana et al. [73] conducted a randomized controlled clinical trial to evaluate autogenous whole-tooth versus DDM grafts for alveolar ridge preservation and they concluded that the two grafts have similar clinical effects but histologically autogenous DDM grafts seems to demonstrate better graft remodeling, integration, and osteoinductive properties. Recently, Ouyyamwongs et al. [76] assessed clinically in a split-mouth randomized controlled clinical trial the potential of using autologous DDM in combination with platelet-rich fibrin (PRF) membrane or PRF membrane alone to preserve the ridge dimension. They concluded that the combination therapy reduced the horizontal ridge collapse, and promoted bone healing as shown clinically and radiographically.

## Discussion

Regenerative dentistry has widely been recognized as a promising field in the provision of functional and biocompatible dental tissues as alternatives for conventional materials. Current progress in tissue engineering has offered new methods and technologies for dentin-pulp complex and bone regeneration. As there are several reasons for dental extraction, including caries, mobility, orthodontic tooth reasons, and trauma, DDM can be prepared with low risks of infection and rejection with non-invasive attainability; thus, it should be considered as a natural resource to be used to full advantage for other applications.

DDM is autogenous tooth dentin that has osteoconductive and osteoinductive potential since dentin contains extracellular COL-1 and various growth factors. Based on the demineralization process, the factors remain available to the host environment; however, extracting proper concentrations of collagen and bioactive molecules from the extracted teeth is a challenging task and requires meticulous preparation of the tooth dentin. DDM is used in dental surgery in the treatment of extraction socket preservation and guided bone regenerations. It functions in a dual capacity: First as a scaffold to support bone regeneration and second as a carrier for bone morphogenic protein (BMP-2). When DDM serves as a carrier, it combines the properties of the grafting material with those of the delivered substances [38].

The type of demineralizing agents and time frames used to prepare DDM are dissimilar in different studies, and therefore they affect the amount of the mineral percentage remaining in the DDM. The DDM components have different inorganic/organic ratios compared to those originally found in the dentin matrix. Approximately, the powder form has mineral content of about 5%–10%, whereas block form has a mineral content of about 10%–30% [1]. ELISA performed on dentin particles showed a slightly higher amount of COL-1 in demineralized samples, as compared with untreated ones, although no significant difference was found and such a finding provides compelling evidence that the demineralization process didn't damage the extracellular matrix of dentin [85]. COL-1 was identified by electrophoresis approximately at 110–120 kDa [86]. Minor bands at 76–102 kDa were detected by electrophoresis, corresponding to dentin matrix protein-1, osteocalcin, osteopontin, proteoglycan, glycoprotein, sialoprotein, and phosphophoryn [87]. In vivo, osteonectin was found in the dentinal tubules of DDM [88]. Besides, the demineralization process is required for freeing the various growth factors and proteins which are essential for tissue regeneration and repair. Consequently, demineralization process is believed to induce release an abundant amount of transforming growth factor-β; an intermediate abundance of BMP-2, fibroblast growth factor-2, vascular endothelial growth factor, platelet-derived growth factor and insulin-like growth factor-1 with a lower abundance from BMP-4 and BMP-7 [89].

The degree of demineralization is critical for optimal dentin regeneration; the partially demineralized dentin matrix (PDDM) is thought to have optimal conditions for dentin regeneration. Partial demineralization results in the elimination of the major part of the mineral phase and immunogenic components while retaining a very low fraction of minerals (5–10 wt%), providing an osteoconductive and osteoinductive scaffold containing several growth factors [62]. On the other hand, the complete DDM (CDDM) showed bone resorption in the early stage of bone regeneration, probably because of the enzymatic digestion of exposed collagen [90]. PDDM probably promotes more osteogenic effects than CDDM does since several noncollagenous proteins were released from the dentin matrix during the process of demineralization. This may account for the more prominent bone formation in PDDM than CDDM in most previous studies.

In terms of particle size, the dentinogenic properties of DDM were greatly affected by the size and shape of dentin matrix particles. The larger-sized particles of the DDM, whose sizes ranged between 350 μm and 800 μm, were found to have better bone regeneration results than the smaller-sized particles that had more resorbability in the defect site before the initiation of new bone formation [89]. PDDM with larger particle sizes induced prominent bone regeneration, probably because PDDM possessed a suitable surface for cell attachment. There might be an exquisite balance between its resorption and bone formation on it. PDDM could be considered as a potential bone substitute.

Dentin and bone are mineralized tissues known to be an organic–inorganic hybrid. They are almost similar.

n their biochemical components but dentin is acellular matrix, while bone includes osteocytes. Autogenous bone is an ideal bone graft material as it has osteoconductive, osteoinductive and osteogenic capabilities but the major drawbacks of autogenous bone are that it requires a secondary donor site with an increase in the susceptibility risk of infection, therefore clinicians prefer commercially available non-autogenous graft materials. Allogenic and xenogeneic bone grafts have osteoinductive ability, but risk of viral infection still remains a considerable problem. Alloplastic bone grafts are clinically used, but they have disadvantages such as limited osteoinductivity and high cost [91]. Among these, demineralized freeze-dried bone allografts have been widely used for bone augmentation [92]. Demineralized bone matrix (DBM) likely as DDM is predominantly composed of COL-1 (95%) with the remainder comprising of NCPs with a small amount of growth factors. Consequently, DDM and DBM can be defined as acid-insoluble collagen bind with BMPs, which are members of the TGF-β super-family that enhances bone formation [93]. The quality and effectiveness of commercial DBM varies with processing techniques and several donor dependent factors likely as gender and age as they have an effect on osteoinductivity of DBM [94]. Therefore, differences in preparation and processing methods for bone can impact properties and clinical performance of DBM. In our opinion, the superiority of autogenous DDM over other graft materials should be confined to the dental applications facilitating the release of BMPs to induce the differentiation of undifferentiated mesenchymal cells into osteogenic and odontogenic cells, which have the potential to stimulate bone and dentin formation [95]. DDM is biocompatible, does not induce foreign body reactions and can be prepared by a standard treatment with very low cost.

It is beneficial to utilize extracted teeth as bone substitutes in implant dentistry, even though there are limited cases with available teeth, in addition to a limited volume. However, there is no risk of transmitting diseases as it is autogenous tissue and no additional surgery is needed to harvest tissues since unwanted teeth are utilized and this raises the need for tooth banking. The Korea Tooth Bank, which was established in Seoul in 2009, is one such tooth-banking facility that can procure and store teeth, and then process them into bone graft substitutes. The Hospital Tooth Bank at Seoul National University Bundang Hospital, which was established in 2010 for performing storage and grafting of auto-tooth bone grafts based on experimental and clinical research.

## Concluding remarks and future perspectives

Before clinical application and for each specific purpose, optimizing protocols of demineralization, characterization, developing a proper consistency, and applying a proper handling technique are necessary for standardization. Further studies are required to determine the most suitable conditions of demineralization and particle sizes for clinical application in implant dentistry.

## Supplementary Information

Below is the link to the electronic supplementary material.

Supplementary file1 (PDF 239 kb)
