# Correction: Demineralized Dentin Matrix for Dental and Alveolar Bone Tissues Regeneration: An Innovative Scope Review

**DOI:** 10.1007/s13770-022-00463-3

**Published:** 2022-05-20

**Authors:** Mohammed E. Grawish, Lamyaa M. Grawish, Hala M. Grawish, Mahmoud M. Grawish, Ahmed A. Holiel, Nessma Sultan, Salwa A. El-Negoly

**Affiliations:** 1 Department of Oral Biology, Faculty of Dentistry, Mansoura University, Elgomhouria St., Mansoura, 35516 Egypt; 2 Faculty of Oral and Dental Medicine, Delta University for Science and Technology, Costal International Road in Front of Industrial Area, Mansoura, 11152 Gamasa Egypt; 3 Mansoura Manchester Dental Program, Faculty of Dentistry, Mansoura University, Elgomhouria St., Mansoura, 35516 Egypt; 4 Department of Conservative Dentistry, Faculty of Dentistry, Alexandria University, 22 El-Guish Road, El-Shatby, Alexandria, 21544 Egypt; 5 Department of Dental Biomaterials, Faculty of Dentistry, Mansoura University, Elgomhouria St., Mansoura, 35516 Egypt

## Correction to: Tissue Eng Regen Med https://doi.org/10.1007/s13770-022-00438-4

In this article the graphics relating to Figs. 3, 4, 5 and 6 captions had been interchanged; the figures should have appeared as shown below.

**Fig. 3:**
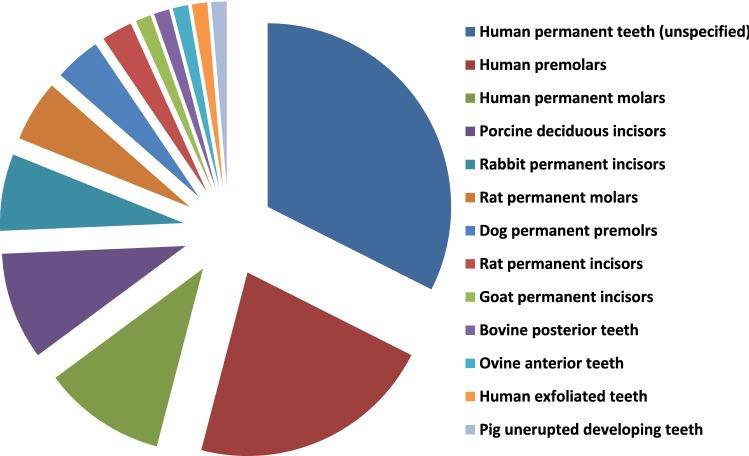
Exploded pie chart showing analytical data of the frequencies regarding source of teeth selected in study designs from the relevant articles

**Fig. 4:**
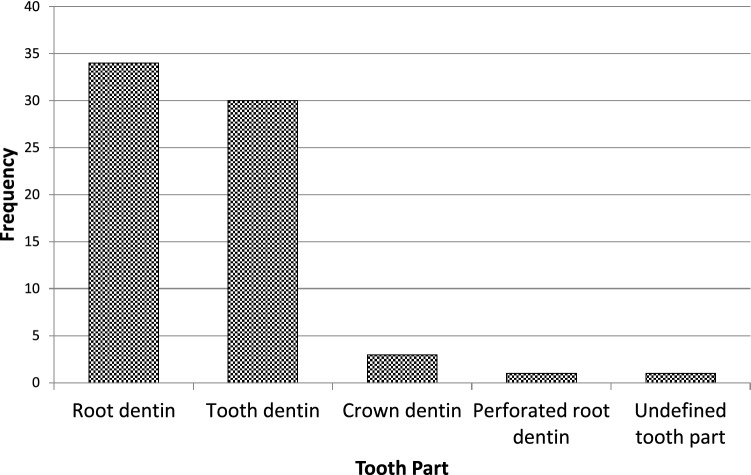
Bar chart showing analytical data of the frequencies regarding tooth part selected in study designs from the relevant articles

**Fig. 5:**
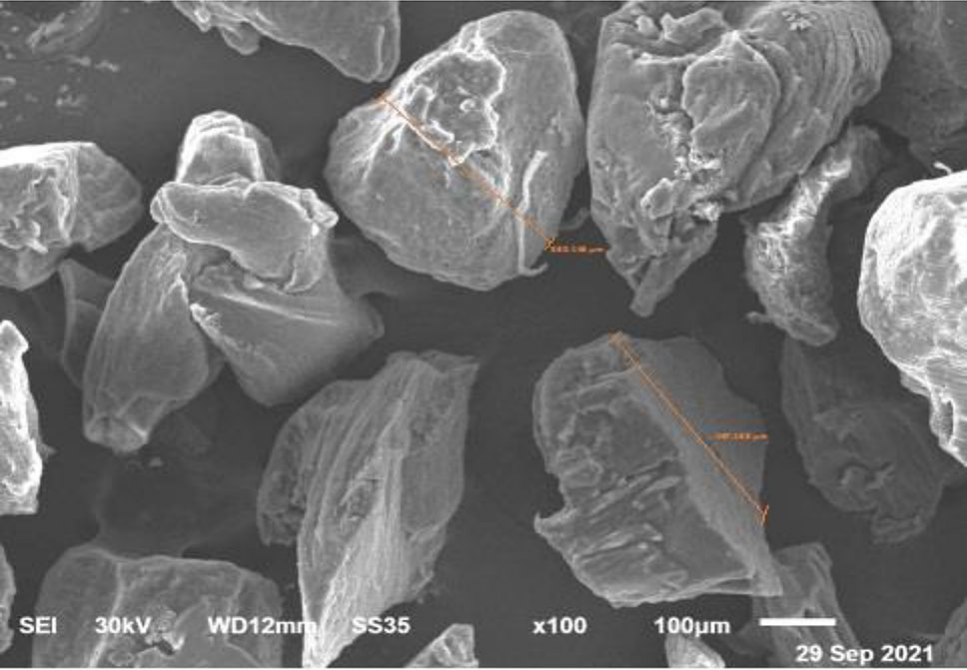
SEM images showing DDM particle size ranging from 350-500lm. Courtesy provided by the staff members of Oral Biology, Faculty of Dentistry, Mansoura University, Mansoura, Egypt

**Fig. 6:**
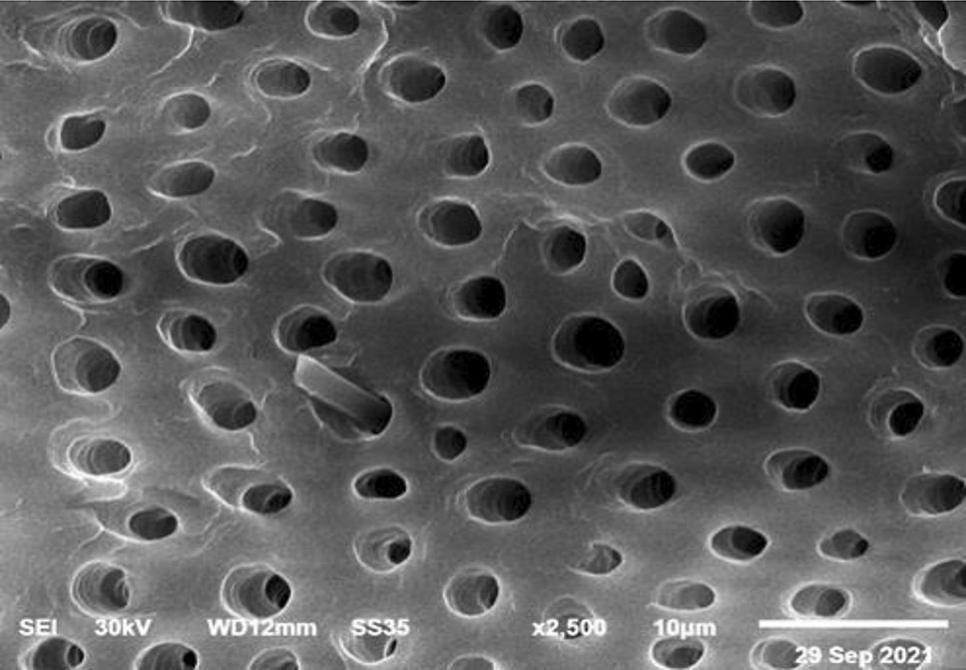
SEM images showing the basic dentin micro-texture after demineralization. Structurally, dentinal tubules are enlarged. Courtesy provided by the staff members of Oral Biology, Faculty of Dentistry, Mansoura University, Mansoura, Egypt.

The original article has been corrected.

