# Supplementary material for: Demineralized Dentin Matrix for Dental and Alveolar Bone Tissues Regeneration: An Innovative Scope Review
Source: Tissue Eng Regen Med. 2022 Apr 16;19(4):687–701. doi: 10.1007/s13770-022-00438-4 (PMC9294090; doi:10.1007/s13770-022-00438-4)
Supplement: Supplementary file 1 — Supplementary file1 (PDF 239 kb) [file 13770_2022_438_MOESM1_ESM.pdf]

Table 1: Study characteristics extracted from PubMed database using TDM and regeneration as search terms.

N	Authors (year, country)	Study design	Source of TDM	Tooth part	Matrix form	Matrix size	Demineralization protocol	Sterilization	Preservation	Outcome
1	Liu et al. (2021, China) [17]	*In-vitro/ in-vivo* animal study	Human teeth	Undefined	Particles	Undefined	17 mol/L EDTA for 10 min, 10 mol/L for 5 min, and 5 mol/L for 10 min	Co-6$_0$ irradiation	Stored at −80 °C	Odontogenic differentiation
2	Li et al. (2021, China) [18]	*In-vitro/ in-vivo* animal study	-Human premolars<br>-Porcine deciduous incisors	Root dentin	Root-shaped matrix	10 mm length and 1.0 mm thickness	-17% EDTA for 5 min, 10% for 5 min, and 5% for 10 min<br>-17% EDTA for 20 min, 10% for 18 min, and 5% for 15 min	PBS supplemented with antibiotics for 72 h	α-MEM at 4 °C	Bio-root regeneration
3	Zhang et al (2021, China) [19]	*In-vitro/ in-vivo* animal study	-Human premolars<br>-Rat molars	Root dentin	-Particles (liquid extract)<br>-Sheet scaffold	Undefined	Gradient concentrations of EDTA	Ethylene oxide	Freeze-dried	Bio-root regeneration
4	Fu et al. (2021, China) [20]	*In-vitro/ in-vivo* animal study	Beagles premolars	Root dentin	Root-shaped matrix to simulate dental pulp cavity	Undefined	17% EDTA for 12 min, 10% for 12 min, and 5% for 20 min	PBS supplemented with antibiotics for 24 h in 37°C	α-MEM at 4 °C	Dental pulp regeneration
5	Holiel. et al. (2021, Egypt) [21]	*In-vivo* human study	Human teeth	Perforated root dentin	Hydrogel	350-500-μm particle-sized powder dispersed in the sodium alginate solution	17% EDTA for 10 min, 10% for 10 min, and 5% for 5 min	PBS supplemented with antibiotics for 72 h	Single syringe	Direct pulp capping
6	Zhang et al. (2020, China) [22]	*In-vitro/ in-vivo* animal study	Human premolars and molars	Root dentin	Liquid extract of hTDM particles	Undefined	17% EDTA for 5 min, 10% for 5 min, and 5% for 10 min	PBS supplemented with antibiotics for 72 h	Undefined	Dental pulp tissue regeneration
7	Chang et al. (2020, Taiwan) [23]	*In-vitro/ In-vivo* animal study	-Human premolars -Goat incisors	Root dentin	Autoclaved sheet scaffold	2 mm × 6 mm × 6 mm dentin matrices	-17% EDTA for 5 min, and 5% EDTA for 5 min	Steam sterilization at 121 ºC and 1 bar for 15 min	Undefined	Tooth regeneration
8	Meng et al. (2020, China) [24]	*In-vitro/ In-vivo* animal study	Human teeth	Root dentin	Disc-shaped	Disc with 5 mm thickness	10% EDTA for 3 days, and then successively soaked in 17% EDTA for 20 min, 10% EDTA for 20 min, and 5% EDTA for 20 min	PBS supplemented with antibiotics for 72 h	α-MEM at 4 °C	Bio-root regeneration
9	Bakhtiar et al. (2020,Iran)	*In-vitro/ In-vivo* animal study	Bovine posterior teeth	Root dentin	Atelopeptidized particles	250- to 500-μm particles	17% EDTA for 1, 7, and 13 days, respectively followed	Frozen	Stored at -20 °C .	Dentin-pulp tissue engineering

	[25]						by atelopeptidization			
**10**	Yang et al. (2019, China) [26]	*In-vitro/ In-vivo* animal study	Human premolars	Root dentin	Particles	Undefined	17% EDTA for 10 min, 10% for 10 min, and 5% for 10 min	Co^{60} radiation sterilization	Stored at -80 °C	Bio-root regeneration
**11**	Yang et al. (2019, China) [27]	*In-vitro/ In-vivo* animal study	Human premolars	Root dentin	Root-shaped matrix	Undefined	17% EDTA for 12 min, 10% for 12 min, and 5% for 20 min	PBS supplemented with antibiotics for 24 h in 37 °C	α-MEM at 4 °C	Bio-root regeneration
**12**	Sun et al. (2017, China) [28]	*In-vitro/ In-vivo* animal study	Porcine deciduous incisors	Root dentin	Root-shaped matrix	-10 mm in length and 3-5 mm in diameter<br>-2 mm in length and 1 mm in diameter	17% EDTA for 20 min, 10% for 18 min, and 5% for 15 min	PBS supplemented with antibiotics for 72 h	α-MEM at 4 °C	Osteoclastogenesis and osteoclastic resorption
**13**	Chen et al. (2017, China) [29]	*In-vitro/ In-vivo* animal study	-Human premolars<br>-Porcine deciduous incisors	Root dentin	Paste	20 g of TDM powder with particles size <76 µm mixed with aqueous TDM extract	17% EDTA for 10 min, 10% for 10 min, and 5% for 5 min	Undefined	Undefined	Dentin regeneration
**14**	Li et al. (2017, China) [30]	*In-vitro/ In-vivo* animal study	-Porcine deciduous incisors	Root dentin	Root-shaped matrix	-10 mm in length and 3-5 mm in diameter	17% EDTA for 20 min, 10% for 18 min, and 5% for 15 min	PBS supplemented with antibiotics for 72 h	α-MEM at 4 °C	Tooth tissue remodeling
**15**	Bakhtiar et al. (2017, Iran) [31]	*In-vitro/ In-vivo* animal study	Dogs premolars	Root dentin	Undefined	2-mm-diameter scaffold	17 % EDTA for 5 min, 10 % for 5 min, and 5 % for 10 min	PBS supplemented with antibiotics for 72 h	α-MEM at 4 °C	Furcation perforation repair
**16**	Wang et al. (2016, China) [32]	*In-vivo* animal study	Human premolars	Root dentin	Root-shaped matrix	1.0 cm length and up to 1.0 mm thickness	17 % EDTA for 5 min, 10 % for 5 min, and 5 % for 10 min	PBS supplemented with antibiotics for 72 h	α-MEM at 4 °C	Periodontal tissue regeneration.
**17**	Tian et al. (2015, China) [33]	*In-vitro/ In-vivo* animal study	Human premolars	Root dentin	Undefined	Undefined	17 % EDTA for 5 min, 10 % for 5 min, and 5 % for 10 min	PBS supplemented with antibiotics for 72 h	DMEM at 4 °C	Dentin tissue regeneration
**18**	Luo et al. (2015, China) [34]	*In-vivo* animal study	Mini-swine Incisors	Root dentin	Tapered shape	The upper diameter 4.9 mm, bottom diameter 3.4 mm; and the length 9.4 mm.	17% EDTA for 20 min, 10% for 20 min, and 5% for 10 min.	PBS supplemented with antibiotics for 72 h	α-MEM at 4 °C	Bio-root regeneration
**19**	Chen et al. (2015, China) [35]	*In-vitro/ In-vivo* animal study	Human premolars	Root dentin	Root-shaped matrix	Undefined	17 % EDTA for 5 min, 10 % for 5 min, and 5 % for 10 min	PBS supplemented with antibiotics for 72 h	DMEM at 4 °C	Dentin tissue regeneration
**20**	Chen et al. (2015, China)	*In-vitro/ In-vivo* animal study	Mini-swine Incisors	Root dentin	Root-shaped	Undefined	17% EDTA for 10 min, 10% for 10 min, and 5% for 5	PBS supplemented with antibiotics for 72	α-MEM at 4 °C	Periodontium and dental pulp

	[36]				matrix		min.	h		regeneration
**21**	Ji et al. (2015, China) [37]	*In-vitro/ In-vivo* animal study	Canine premolars	Root dentin	Root-shaped matrix	undefined	17% EDTA for 8 min, 10% for 8 min, and 5% for 12 min	undefined	Undefined	Tooth root regeneration
**22**	Jiao et al. (2015, China) [38]	*In-vitro/ In-vivo* animal study	Human premolars	Root dentin	Root-shaped matrix	length of 1.0 cm and a diameter of 4 mm	17 % EDTA for 5 min, 10 % for 5 min, and 5 % for 10 min	PBS supplemented with antibiotics for 72 h	cryopreserved in liquid nitrogen (−196 °C)	Dentin-pulp tissue regeneration
**23**	Yang et al. (2014, China) [39]	*In-vitro/ In-vivo* animal study	Rat molars	Root dentin	Undefined	Undefined	17% EDTA for 3-4 min, and 5% for 2 min	PBS supplemented with antibiotics for 72 h	α-MEM at 4 °C	Osteogenic differentiation
**24**	Guo et al. (2013, China) [40]	*In-vitro/ In-vivo* animal study	Human premolars	Root dentin	Undefined	Undefined	17% EDTA, then in 10% and finally in 5% (time undefined)	PBS supplemented with antibiotics for 72 h	α-MEM at 4 °C	Odontogenic Differentiation
**25**	Yang et al. (2012, China) [41]	*In-vitro/ In-vivo* animal study	Human premolars	Root dentin	Root-shaped matrix	1.0 cm length and up to 1.0 mm thickness	17 % EDTA for 5 min, 10 % EDTA for 5 min, and 5 % EDTA for 10 min	PBS supplemented with antibiotics for 72 h	α-MEM at 4 °C	Tooth root regeneration
**26**	Guo et al. (2012, China) [42]	*In-vivo* animal study	Rat molars	Root dentin	Sheet scaffold	2 mm × 6 mm × 6 mm dentin matrices	17% EDTA for 3-4 min, and 5% for 2 min	PBS supplemented with antibiotics for 72 h	α-MEM at 4 °C	Tooth roots regeneration
**27**	Li et al. (2011, China) [43]	*In-vitro*/ *in-vivo* animal study	Human premolars	Root dentin	Root-shaped scaffold	Undefined	17 % EDTA for 5 min, 10 % for 5 min, and 5 % for 10 min	PBS supplemented with antibiotics for 72 h	DMEM at 4 °C	Dentin regeneration
**28**	Guo et al. (2009, China) [44]	*In-vitro*/ *in-vivo* animal study	Rat molars	Root dentin	Sheet scaffold	2 mm × 6 mm × 6 mm dentin matrices	17% EDTA for 3-4 min, and 5% for 2 min	PBS supplemented with antibiotics for 72 h	α-MEM at 4 °C	Dentin regeneration

Table 2: Study characteristics extracted from PubMed database using DDM and regeneration as search terms

N	Authors (year, country)	Study design	Source of TDM	Tooth part	Matrix form	Matrix size	Demineralization protocol	Sterilization	Preservation	Outcome
**1**	Kim et al. (2021, Republic of Korea) [45]	*In-vivo* animal study	Human teeth	Root dentin	Pulverized particles	1–2 mm pulverized particles	0.6 N HCl	Ethylene oxide gas	Room temperature	Bone regeneration
**2**	Ku et al. (2021, Republic of Korea) [46]	Case report	Human molars	Root dentin	Barrier Membrane	300 to 800 µm thick slice of autogenous tooth bone graft block with 0.2 to 0.3 mm diameter holes	0.6 N HCl	Freeze-drying	Packaged	Guided bone regeneration
**3**	Han et al. (2021, Republic of Korea) [47]	*In-vitro study*	Human teeth	Tooth dentin	Particle-Based Bio-Ink	≤100 µm	4 weeks with a 0.5 M EDTA containing protease inhibitors	Gradient ethanol	Undefined	3D dental tissue regeneration
**4**	Kabir et al. (2021, Japan) [48]	*In-vivo* animal study	Human molars	Tooth dentin	Perforated root-demineralized dentin matrix	The average length and width were 13.08 and 8.41 mm, respectively	supersonic demineralization in 5 L of 0.34 N HNO_3 solutions at 100 W and 60 kHz for 30 min	Undefined	Undefined	Critical-size Iliac defects' regeneration
**5**	Jing et al. (2021, China) [49]	*In-vitro/ in-vivo* animal study	Human teeth	Tooth dentin	Decorated particles via carboxymethyl chitosan	100-700 µm in diameter	17 mol L−1 (5 min), 10 mol L-1 (5 min), 5 mol L-1 (10 min) EDTA	Ethylene oxide gas	stored at 4 °C	Osteogenic differentiation and bone regeneration
**6**	Gao et al. (2020, China) [50]	*In-vitro* study	Human teeth	Tooth dentin	Particles	≤ 300−1000 µm	0.71 M HCl for 12 h at 37 °C in a shaker	Undefined	0.1× PBS at 4 °C	Osteogenic differentiation
**7**	Umebayashi et al. (2020, Japan) [51]	Case report	Human teeth	Tooth dentin	Particles of partially DDM and Particulate Cancellous Bone and Marrow	A diameter of approximately 1 mm	Partially demineralized in 2% HNO_3	Undefined	Prepared during the operation for bone augmentation	Full regeneration of maxillary alveolar bone
**8**	Um et al. (2019, Republic of Korea) [52]	Case Series	Human teeth	Root dentin	Particles loaded with recombinant human bone morphogenetic-2	300-800 µm in diameter	Demineralized for 30 min in 0.6 N HCl	Ethylene oxide gas	stored at room temperature	Socket preservation

**9**	Li et al. (2018, China) [53]	Prospective clinical study	Human teeth	Tooth dentin	Granules	300 -1200 µm	Demineralized in 2% HNO_3 for 20 minutes	5% peracetic acid and 75% ethanol for 10 min	Freshly prepared	Guided bone for immediate implantation regeneration
**10**	Melling et al. (2018, UK) [54]	*In-vitro* study	Human teeth	Tooth dentin	DDM extract-loaded liposomes	Liposomes of ~100 nm ±20 nm	7.5 % EDTA diNa+	Lyophilized	undefined	Dental tissue regeneration
**11**	Minamizatoet al. (2018, Japan) [55]	Prospective clinical study	Vital and non-vital human teeth	Tooth dentin	Particles	400 µm to 800 µm	2% HNO_3 (pH 1.0) for 10 min	Rinsed extensively twice in 0.1 M Tris–HCl (pH 7.4) for 10 min	Freshly prepared	Bone regeneration procedures related to implant dentistry, including socket preservation, alveolar ridge augmentation, and maxillary sinus floor augmentation
**12**	Kabir et al. (2017, Japan) [56]	*In-vivo* animal study	Human vital non-functional wisdom teeth	Tooth dentin	Perforated root-demineralized dentin matrix	Thirty pores of 1mm hole, as the pore size	Supersonic demineralization in 0.34N HNO_3 solution at 100W and 60kHz for 30min	Undefined	Undefined	Bone regeneration in critical-size sheep iliac defects
**13**	Pang et al. (2017, Republic of Korea) [57]	prospective randomized clinical trial	Human teeth	Tooth dentin	Particles	300 and 800 µm	0.6 N HCl	Ethylene oxide gas	Room temperature	Alveolar bone defect
**14**	Nam et al. (2016, Republic of Korea) [58]	*In-vivo* animal study	Human teeth	Tooth dentin	Particles	0.25 to 1.0 mm in size or 1.0 to 2.0 mm in size	Demineralized for 15 h in hydrochloric acid	Ethylene oxide gas	Packaged	Cranial bone regeneration
**15**	Kim et al. (2016, Republic of Korea) [59]	*In-vivo* animal study	Human teeth	Tooth dentin	Particles	1~2-mm-sized crushed particles	Undefined	Ethylene oxide gas	Packaged	Bone-forming capacity
**16**	Kim et al. (2016, Republic of Korea) [60]	Case report	Human teeth	Tooth dentin	Particles	0.5 to1.0 mm	Undefined	Ethylene oxide gas	Packaged	Guided Bone Regeneration
**17**	Gomes et al. (2016, Brazil) [61]	*In-vivo* animal study	Central incisors of the normoglycaemic rabbits	Tooth dentin	Slices	8 µm in thickness	0.6 N-HCl solution at 2°C until complete demineralization	Slices were immersed in a box filled with 5 mL alcohol/0.2 mL of gentamicin	Stored at 2°C until implantation	Bone tissue engineering in cranioplasty of diabetic rabbits
**18**	Koga et al. (2016, Japan) [62]	*In-vitro/ in-vivo* animal study	Vital or non-vital human teeth	Tooth dentin	Particles	180 µm/ 1200 µm	2% HNO_3, partially/ completely demineralized dentin matrix	Rinsed twice in 0.1 M Tris-HCl (pH 7.4), for 10 minutes.	Undefined	Bone Regeneration
**19**	Liu et al. (2016, China) [63]	*In-vitro/ in-vivo* animal study	Human teeth	Tooth dentin	Extract	1 mg/ 10 mg/ml	Undefined	Undefined	Freshly prepared	Odontoblastic Differentiation of Dental Pulp Stem

										Cells
**20**	Qin et al. (2015, China) [64]	*In-vitro*/ *in-vivo* animal study	Human teeth	Crown dentin	Semi-rigid barrier	Slices of cubic appearance with 2 × 2 × 8 mm	0.6 M HCl solution	Undefined	Undefined	Guiding periodontal tissue regeneration
**21**	Bakhshalian et al. (2013, USA) [65]	*In-vivo* animal study	Rabbit mandibular incisor	Tooth dentin	Particles	300 µm	0.6 M HCl for 1 week/ tooth	The teeth were mixed in a sterile container	Room temperature	Osteopromotive Property
**22**	Li et al. (2013, China) [66]	*In-vitro*/ *in-vivo* animal study	Human teeth	Tooth dentin	Granules	10–140 meshes	Undefined	irradiated with Cobalt 60 at a radiation dose of 5 kGy	Undefined	Bone tissue regeneration
**23**	Zhang et al. 2012, China) [67]	*In-vitro*/ *in-vivo* animal study	Incisors of twenty 8-week-old SD rats	Tooth dentin	Granules	200– 280 µm	17% EDTA, pH 8.0 at 4C for 10 min	Ethylene oxide gas	Undefined	Dentinogenic Potential of Dental Pulp Stem Cells
**24**	Yagihashi et al. (2009, Japan) [68]	*In-vivo* animal study	ovine lower anterior teeth	Tooth dentin	Particles	250- to 500-µm	0.6 N HCl for 1 week/ tooth	Ethylene oxide gas	Undefined	Repair of articular cartilage defects
**25**	Gomes et al. (2008, Brazil) [69]	*In-vivo* animal study	Central incisors of the normoglycaemic rabbits	Tooth dentin	Slices	8 µm in thickness	0.6 N-HCl solution at 2°C until complete demineralization	Slices were immersed in a box filled with 5 mL alcohol/0.2 mL of gentamicin	Stored at 2°C until implantation	Bone repair
**26**	Gomes et al. (2002, Brazil) [70]	*In-vivo* animal study	Central incisors of the normoglycaemic rabbits	Tooth dentin	Slices	8 µm in thickness	0.6 N-HCl solution at 2°C until complete demineralization	Slices were immersed in a box filled with 5 mL alcohol/0.2 mL of gentamicin	Stored at 2°C until implantation	Bone repair
**27**	Gomes et al. (2001, Brazil) [71]	*In-vivo* animal study	Central incisors of the normoglycaemic rabbits	Tooth dentin	Slices	8 µm in thickness	0.6 N-HCl solution at 2°C until complete demineralization	Slices were immersed in a box filled with 5 mL alcohol/0.2 mL of gentamicin	Stored at 2°C until implantation	Bone repair

Table 3: Study characteristics extracted from Medline (OVID) database using TDM and regeneration as search terms.

N	Authors (year, country)	Study design	Source of TDM	Tooth part	Matrix form	Matrix size	Demineralization protocol	Sterilization	Preservation	Outcome
**1**	Xu et al. (2021, China [72]	*In-vitro*/ *in-vivo* animal study	Mandibular and maxillary premolars	Root dentin	Root segment	10 mm-long segments, and the root canal was enlarged to 5 mm in diameter of both ends	17%, 10%, and 5% EDTA, respectively, for 30 minutes	Soaked into penicillin-streptomycin	4ºC over 3 days	Dental Pulp Regeneration
**2**	Elfana et al. (2021, Egypt) [73]	A randomized controlled clinical trial	Non-molar human teeth	Tooth dentin	Particles	Undefined	0.6 N HCl for 30 min	Undefined	Undefined	Alveolar ridge preservation
**3**	Wen et al. (2021, China) [74]	*In-vitro*/ *in-vivo* animal study	-Healthy human third molars<br>-Porcine incisors	Tooth dentin	Particles to get aqueous extracts	≤ 40 μm	17%, 10%, and 5% EDTA, respectively, for 30 minutes	Sterilized by radiation of Co_{60}	α-MEM culture medium for 7 days	Reparative dentin formation
**4**	Mehrvarzfar et al. (2018, Iran) [75]	Clinical trial	Intact human third molars	Crown dentin	Dentin chips	1 mm thickness	17% EDTA for 5 min, 10% EDTA for 5 min and 5% EDTA for 10 min	Rinsed with sterile saline for 10 min	stored in PBS until required	Partial pulpotomy

Table 4: Study characteristics extracted from Medline (OVID) database using DDM and regeneration as search terms.

N	Authors (year, country)	Study design	Source of TDM	Tooth part	Matrix form	Matrix size	Demineralization protocol	Sterilization	Preservation	Outcome
1	Ouyyamwongs et al. (2019,Thailand) [76]	A Split-Mouth Randomized Controlled Clinical Trial	Intact human third molars	Tooth dentin	Particles	500-700 µm	Undefined	Sterilized with ethylene oxide gas	Undefined	Alveolar Ridge Preservation
2	Xu et al. (2018, Republic of Korea) [77]	*In-vivo* animal study	Human permanent teeth	Tooth dentin	Particles	0.8 to 1.0 mm	0.6N HCl for 15 min	Sterilized with sterilization reagent	Undefined	Maxillary sinus augmentation
3	Feng et al. (2018, China) [78]	*In-vitro*/ *in-vivo* animal study	Intact human third molars	Root dentin	Porous sheet	100 µm thickness	0.6 M HCl for 2 weeks	Dipped in PBS and placed at 4°C overnight	Put in a sterile EP tube at−20°C until use	Periodontal ligament-like tissue regeneration
4	Al-Asfour et al. (2017, Sweden) [79]	*In-vivo* animal study	Human premolars	Root dentin	Cylindrical dentin block	5 mm in diameter and 2 mm thickness	24%EDTA neutral, pH7, for 12 h	Undefined	Undefined	Onlay grafts to rabbit tibia
5	Park et al. (2015, Republic of Korea) [80]	*In-vivo* animal study	Exfoliated deciduous teeth	Crown dentin	Particles	800 -1000 µm in size	0.6N HCl was done for 0, 10, 15, 20, 25, 30, 60and 90 min respectively for *in vitro* analysis, and for 15 min for *in vivo* experiment	Sterilized with peracetic acid-ethanol solution	Prepared sample was kept in the refrigerator at 4°C	Bone healing
6	Park et al. (2015, Republic of Korea) [81]	*In-vitro*/ *in-vivo* animal study	Extracted human permanent teeth	Tooth dentin	Dentin blocks	5 mm in diameter, 2 mm in height	0.6N HCl for 10 and 60 min each	Sterilized with peracetic acid-ethanol solution	Prepared sample was kept in the refrigerator at 4°C	Bone formation
7	de Oliveira et al. (2013, Brazil) [82]	*In-vivo* animal study	Human permanent teeth	Root dentin	Slice	Undefined	10%EDTA, pH 7.2 at room temperature for approximately 3 months	Washed in sterilized deionized water for 10 min	Stored in sterile PBS containing 100 units/ml penicillin and 100 mg/ml streptomycin	Bone formation during healing process of tooth sockets
8	Mordenfeld et al. (2011, Sweden) [83]	*In-vivo* animal study	Unerupted developing pig teeth	Tooth dentin	Dentin block	2–3mm thick and 4 mm in diameter	24% EDTA (pH 7.0) for 1, 2, 6 or 12 h, respectively, in room temperature	Undefined	Stored for one to 2months in a freezer at -18˚C before implantation	Tissue reactions to subperiosteal onlays
9	Koike et al. (2005, Japan) [84]	*In-vivo* animal study	Rat incisors	Root dentin	Dentin tubes	8 mm in length	0.6 N HCl at room temperature for 3 h	Undefined	Undefined	Ectopic bone formation
